# Making a Better Home: Modulation of Plant Defensive Response by *Brevipalpus* Mites

**DOI:** 10.3389/fpls.2018.01147

**Published:** 2018-08-15

**Authors:** Gabriella D. Arena, Pedro L. Ramos-González, Luana A. Rogerio, Marcelo Ribeiro-Alves, Clare L. Casteel, Juliana Freitas-Astúa, Marcos A. Machado

**Affiliations:** ^1^Laboratório de Biotecnologia, Centro de Citricultura Sylvio Moreira, Instituto Agronômico de Campinas, Cordeirópolis, Brazil; ^2^Instituto de Biologia, Universidade Estadual de Campinas, Campinas, Brazil; ^3^Laboratório de Bioquímica Fitopatológica, Instituto Biológico, São Paulo, Brazil; ^4^Instituto Nacional de Infectologia Evandro Chagas, Fundação Oswaldo Cruz, Rio de Janeiro, Brazil; ^5^Department of Plant Pathology, University of California, Davis, Davis, CA, United States; ^6^Embrapa Mandioca e Fruticultura, Cruz das Almas, Brazil

**Keywords:** plant–herbivore interaction, plant hormones, defense pathways, salicylic acid, jasmonic acid, cross-talk, *Tetranychus*, RNA-Seq

## Abstract

False-spider mites of the genus *Brevipalpus* are highly polyphagous pests that attack hundreds of plant species of distinct families worldwide. Besides causing direct damage, these mites may also act as vectors of many plant viruses that threaten high-value ornamental plants like orchids and economically important crops such as citrus and coffee. To better understand the molecular mechanisms behind plant-mite interaction we used an RNA-Seq approach to assess the global response of *Arabidopsis thaliana* (Arabidopsis) plants along the course of the infestation with *Brevipalpus yothersi*, the main vector species within the genus. Mite infestation triggered a drastic transcriptome reprogramming soon at the beginning of the interaction and throughout the time course, deregulating 1755, 3069 and 2680 genes at 6 hours after infestation (hai), 2 days after infestation (dai), and 6 dai, respectively. Gene set enrichment analysis revealed a clear modulation of processes related to the plant immune system. Co-expressed genes correlated with specific classes of transcription factors regulating defense pathways and developmental processes. Up-regulation of defensive responses correlated with the down-regulation of growth-related processes, suggesting the triggering of the growth-defense crosstalk to optimize plant fitness. Biological processes (BPs) enriched at all time points were markedly related to defense against herbivores and other biotic stresses involving the defense hormones salicylic acid (SA) and jasmonic acid (JA). Levels of both hormones were higher in plants challenged with mites than in the non-infested ones, supporting the simultaneous induction of genes from both pathways. To further clarify the functional relevance of the plant hormonal pathways on the interaction, we evaluated the mite performance on Arabidopsis mutants impaired in SA- or JA-mediated response. Mite oviposition was lower on mutants defective in SA biosynthesis (*sid2*) and signaling (*npr1*), showing a function for SA pathway in improving the mite reproduction, an unusual mechanism compared to closely-related spider mites. Here we provide the first report on the global and dynamic plant transcriptome triggered by *Brevipalpus* feeding, extending our knowledge on plant-mite interaction. Furthermore, our results suggest that *Brevipalpus* mites manipulate the plant defensive response to render the plant more susceptible to their colonization by inducing the SA-mediated pathway.

## Introduction

Plants are frequently threatened by arthropods herbivores from different feeding guilds causing variable tissue injuries. Chewers consume a significant amount of plant tissue thus promoting extensive damage, while sap-suckers and cell-content-feeders pierce to ingest plant fluids, inflicting minimal physical damage. To further enhance self-protection against attackers, plants display receptors that recognize conserved molecules associated with herbivores (herbivore-associated molecular patterns – HAMPs) or even self-molecules released after cell damage inflicted by the attack (damage-associated molecular patterns – DAMPS) and mount appropriate defense response. Some adapted herbivores have evolved the ability to counteract plant defenses by producing effectors that disrupt plant signaling and induce effector-triggered susceptibility (Hogenhout and Bos, 2011; Ferrari et al., 2013; Pel and Pieterse, 2013). The plant counterattack involves resistance proteins (R proteins) which directly bind the effectors, or the plant proteins they modify, and elicit a second layer of the immune response. The outcome of induced defenses includes the production of toxins that interfere with herbivore feeding, growth, reproduction or fecundity and/or volatile compounds that attract natural enemies of the attacker (Pieterse et al., 2012).

Upon recognition a cascade of phytohormone-dependent signals, modulated by the nature of the damage, orchestrates specific plant defense responses. Generally, arthropods such as chewing insects that greatly damage the plant tissue integrity trigger the jasmonic acid (JA) pathway, whilst herbivores causing minimal tissue disruption, i.e., piercing-sucking arthropods induce salicylic acid (SA) mediated response (Arimura et al., 2011). The SA pathway is typically associated with resistance against biotrophic pathogens and can often antagonize JA-mediated defenses. Ethylene (ET) and abscisic acid (ABA) also control plant responses to herbivore through the modulation of JA signaling branches. ABA regulates the MYC transcription factor branch (MYC-branch) acting in the defenses against herbivores, whereas ET regulates the ethylene responsive factor branch (ERF-branch) to defend against necrotrophic invaders. The ET- and ABA-regulated branches antagonize each other to fine tune JA pathway against the specific invader (Pieterse et al., 2012).

Herbivores can take advantage of the natural cross-talk between hormonal pathways to circumvent plant defenses. *Bemisia tabaci* activates SA responses to suppress effective JA defenses and improve whitefly performance (Zarate et al., 2007; Zhang et al., 2013). Likewise, some insect eggs induce high levels of SA that leads to reduced protein levels of MYC2, subsequent suppression of JA defenses, and the enhancement of larvae performance (Bruessow et al., 2010; Schmiesing et al., 2016). The ERF-MYC branch antagonism is also occasionally exploited by herbivores. Oral secretions of *Pieris rapae* activates the ERF-branch to rewire JA signaling toward the insect preferred branch (Verhage et al., 2011). Beyond through cross-talk, other herbivores are capable of directly suppressing several defense pathways. The mite *Tetranychus evansi* repress both JA and SA signaling in tomato, dramatically reducing the levels of defense compounds (Sarmento et al., 2011; Alba et al., 2015).

Manipulation of plant defenses by herbivores has been shown to frequently occur through saliva-contained effectors. Salivary proteins able to modulate defenses and improve herbivore performance have been identified in insects (Hogenhout and Bos, 2011) and mites (Villarroel et al., 2016). Moreover, proteins from arthropod-associated microorganisms such as endosymbiont bacteria (Casteel et al., 2012; Chung et al., 2013) and viruses (Casteel et al., 2014; Li et al., 2014) may also be present in the saliva and modulate plant defenses to promote herbivore performance.

Current understanding of the mechanisms involved in plant response to herbivores comes mainly from studies of plant–insect interactions. Relatively little is known about molecular responses to other arthropods as mites, most of them focusing on the two-spotted spider mite *T. urticae* (Rioja et al., 2017). False spider mites of the genus *Brevipalpus* (Acari: Tenuipalpidae) are economically important phytophagous mites that attack hundreds of plant species of very distinct families, including large-scale plantations of high-value crops and several ornamental plants (Childers et al., 2003; Kitajima et al., 2010). Besides causing direct damage to some plant species, the negative impacts of infestation are often exacerbated by their ability to vector numerous plant-infecting viruses, the so-called *Brevipalpus*-transmitted viruses (BTVs) (Kitajima and Alberti, 2014). *Brevipalpus yothersi* vectors both cileviruses and tentative dichorhaviruses (Ramos-González et al., 2016; Chabi-Jesus et al., 2018) being the main vector of citrus leprosis virus C (CiLV-C), the prevalent virus causing citrus leprosis disease. Chemical control of *B. yothersi* mites costs millions of dollars each year in Brazil, the world leading producer of sweet orange juice, frozen concentrated orange juice (FCOJ) and not-from-concentrate orange juice (NFC) (Bastianel et al., 2010). The cosmopolitan distribution of *Brevipalpus* spp. poses a major threat to the worldwide citrus industry and to other crops such as coffee (Rodrigues and Childers, 2013; Beard et al., 2015). In addition to their agricultural relevance, *Brevipalpus* mites are also prominent because of their unusual biology. Several species of the genus are haploid during their entire life cycle, an exclusive feature amongst higher organisms, and are essentially female due to the presence of the endosymbiont bacterium *Cardinium* sp. (Weeks et al., 2001).

Despite the economic and biological significance, many aspects of the *Brevipalpus* mite-plant interaction remain largely unknown. A previous study showed that plants respond to the presence of *B. yothersi* non-viruliferous mites with a ROS burst and induction of specific genes from SA and JA-dependent pathways (Arena et al., 2016). Upon infestation with CiLV-C viruliferous mites, both SA- and JA-responsive genes are reduced, and mite behavior is affected (Arena et al., 2016). To achieve a wider understanding of the molecular mechanisms behind plant-mite interaction, we used an RNA-Seq approach assessing the global response of *Arabidopsis thaliana* plants along the course of the infestation with *B. yothersi.* Transcriptome analysis was complemented with the measuring of the SA and JA hormone levels in plants upon mite feeding. Finally, to further clarify the functional relevance of hormone-triggered plant defense on the interaction, we evaluated the *B. yothersi* oviposition on Arabidopsis mutants impaired in SA or JA-mediated response. Current work provides a comprehensive picture of the plant response to *Brevipalpus* mite feeding.

## Results

### *Brevipalpus* Mites Elicit a Significant Transcriptome Reprogramming on Infested Plants

**FIGURE 1:**
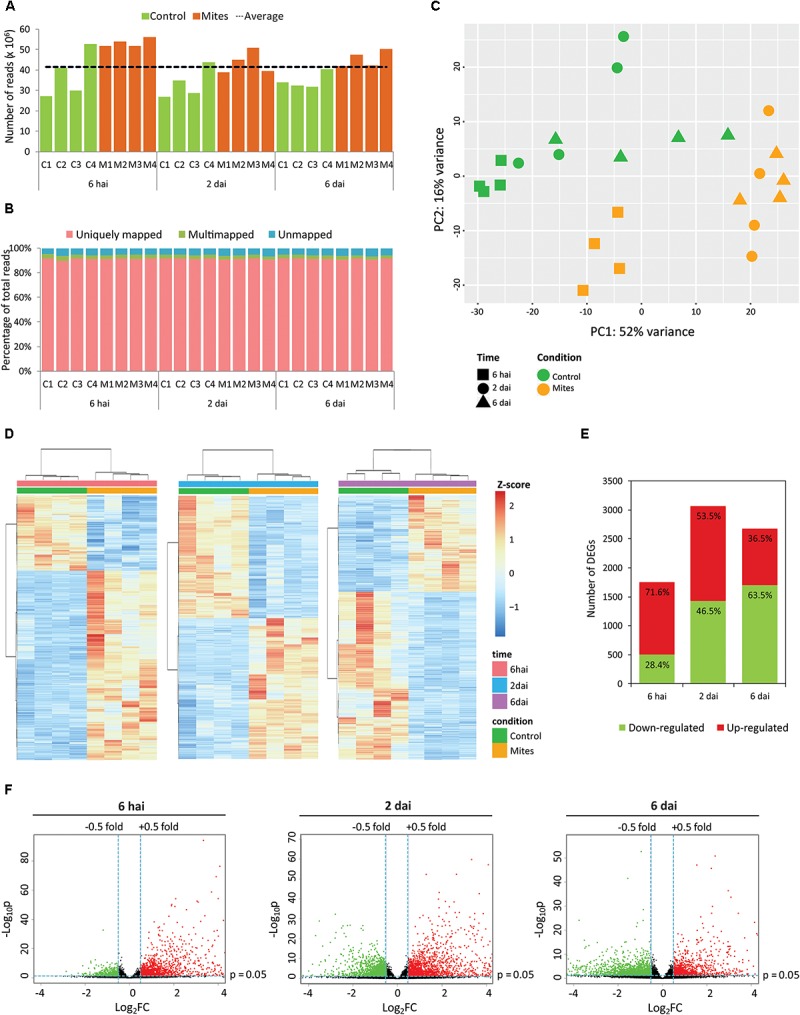
Overview of *Arabidopsis thaliana* transcriptome upon infestation by *Brevipalpus* mites. **(A)** Number of *paired-end* reads generated for each library by Illumina HiSeq sequencing. C, control (non-infested plants); M, mite-infested-plants. Dashed line represents the average of *paired-end* reads from all 24 libraries. **(B)** Proportion of uniquely mapped, multi-mapped and unmapped reads obtained for each library. Reads were mapped in the *A. thaliana* (TAIR 10) genome using *TopHat2*. C, control; M, mite-infected plants. **(C)** Principal component analysis of normalized count data from all samples. **(D)** Hierarchical clustering analysis of normalized count data *z*-scores exhibited by differentially expressed genes (DEGs) of each sample within each time point. **(E)** Numbers of up- and down-regulated DEGs in mite-infested plants in comparison to non-infested control at each time point. DEGs were identified using *DESeq2* and defined by log_2_ fold-change ≥ 0.5 and false discovery rate (FDR)-corrected *p*-value ≤ 0.05. **(F)** Volcano-plots of -log_10_p and log_2_FC exhibited by each gene in mite-infested plants compared to non-infested control at each time point. Up- and down-regulated genes are presented in red and green, respectively. FC, fold-change; p, FDR-corrected *p*-value, hai, hours after infestation; dai, days after infestation.

A time-course RNA-Seq experiment was set up to assess the global response of *A. thaliana* plants along the course of infestation with non-viruliferous *B. yothersi* mites. The transcriptome of infested plants was compared with that from non-infested ones (control) at 6 h after infestation (hai), 2 and 6 days after infestation (dai). Overall, 995 million paired-end reads were obtained by Illumina sequencing, with an average of 41.5 million per library and higher average number of reads in samples from the infested treatment (**Supplementary Table S1** and **Figure 1A**). Roughly 94% of the reads mapped against the *A. thaliana* reference genome, with a 91% average of uniquely mapped reads (**Supplementary Table S1** and **Figure 1B**).

Biological variability between samples was verified by principal component analysis (PCA) using the normalized count data (**Figure 1C**). Infested and control samples grouped separately, suggesting a globally distinct expression profile, as expected. Even though a classification of the first principal components as treatment or time of infestation was not clear, the first component (PC1), which accounts for 52% of the variance, apparently separated the samples by the intensity of stimuli. Except for two out of four control samples at 6 dai, all control samples grouped together with those of mite-infested treatments from 6 hai, whose plants were stimulated by a short period of mite feeding. Samples from plants challenged by longer mite feeding period (2 and 6 dai) grouped separately. Hierarchical clustering of samples within each time point confirmed a clear separation between mite-infested and non-infested treatments over the course of the experiment (**Figure 1D**).

By using the negative binomial-based DESeq2 package and FDR correction of *p*-values for multiple comparisons, 5005 differentially expressed genes (DEGs, α ≤ 0.05) were detected (**Supplementary Table S2**). Mite infestation deregulated 1755, 3069 and 2680 genes at 6 hai, 2 dai, and 6 dai, respectively (**Figure 1E**). At the earliest stage of the interaction (6 hai), the majority of the DEGs was up-regulated. The number of down-regulated genes progressively increased during the interaction reaching its highest rating at 6 dai (**Figures 1E,F**). Analysis performed here show an intense modulation of the plant transcriptome in response to *Brevipalpus* mite infestation.

To validate the RNA-Seq data, 10 genes were selected for Real Time RT-qPCR analysis. Expression of these genes was assessed in a new experiment with mite-infested and non-infested Arabidopsis plants at 6 hai, 2 dai, and 6 dai (**Supplementary Figure S1**). Altogether, expression profiles of selected genes obtained by RT-qPCR were consistent with those obtained by the RNA-Seq, supporting the results described in this work. Additionally, some of these genes had an expression profile similar to that revealed by a qPCR-driven analysis during a comparable experiment previously described (Arena et al., 2016).

### The Plant Immune System Is Modulated by *Brevipalpus* Mite Infestation

Gene ontology (GO) enrichment analysis was performed with all 5005 DEGs to identify the most relevant biological processes (BPs), molecular functions (MFs) and cellular components (CC) disturbed during *Brevipalpus* mite-plant interaction. This study identified 264 BPs, 83 MFs and 78 CCs that were over-represented (hypergeometric test, α ≤ 0.001) in the list of DEGs (**Supplementary Table S3**). Enriched BPs were further visualized as a network using the app BinGO from Cytoscape, where color and size of the nodes identify *p-*values and number of DEGs from each category, respectively (**Supplementary Figure S2**).

The GO network revealed a striking deregulation of plant defensive responses. BP categories were clustered in two major groups comprising metabolism and response to stimuli. BP-metabolism was sub-clustered into two branches separately harboring the primary and secondary metabolisms. Secondary metabolism group was represented by processes related to the biosynthesis and metabolism of “flavonoids,” “glucosinolates,” “toxins,” and “camalexins,” which are known to exert anti-herbivory roles and be induced by SA or JA. Primary metabolism included categories associated to the metabolism of: (i) “aminoacids” and “proteins,” connected to processes involved in the control of gene expression (such as “protein modification,” “phosphorylation,” and “transcription”); (ii) “organic acid,” whose sub-categories included the “SA metabolism” and “JA biosynthesis and metabolism”; and (iii) “carbohydrates,” edged to several photosynthesis-associated categories and processes related to “cell wall modification” such as “callose deposition,” a well-known defense against herbivores (Jander, 2014).

Biological processes cluster centralized in “response to stimulus” was fully represented by processes associated with defense responses. Nodes from the cluster included response to “stress,” “abiotic,” and “biotic” stimulus (linked to “defense response” and to the subcategories of response to “wounding,” “insects,” and other pathogens). A large “response to hormone” branch displayed all main hormone-mediated pathways, including SA and JA, but also abscisic acid (ABA), ethylene (ET), auxins (IAA), cytokinins (CK) and gibberellins (GA). Other nodes included in the “response to stimuli” group were “response to chitin,” commonly triggered in plants colonized by chitin-rich organisms such as arthropods and fungi (Libault et al., 2007), and “oxidative stress,” typically induced in plant-biotic interactions (Arena et al., 2016; Camejo et al., 2016). Several processes related to defense response were also present in “biological regulation” nodes, such as “regulation of hormone levels,” “defense response,” “immune response,” and, “JA pathway.”

### Specific and Common Transcriptomic Changes Occur at Different Time Points After *Brevipalpus* Mite Infestation of Plants

**FIGURE 2:**
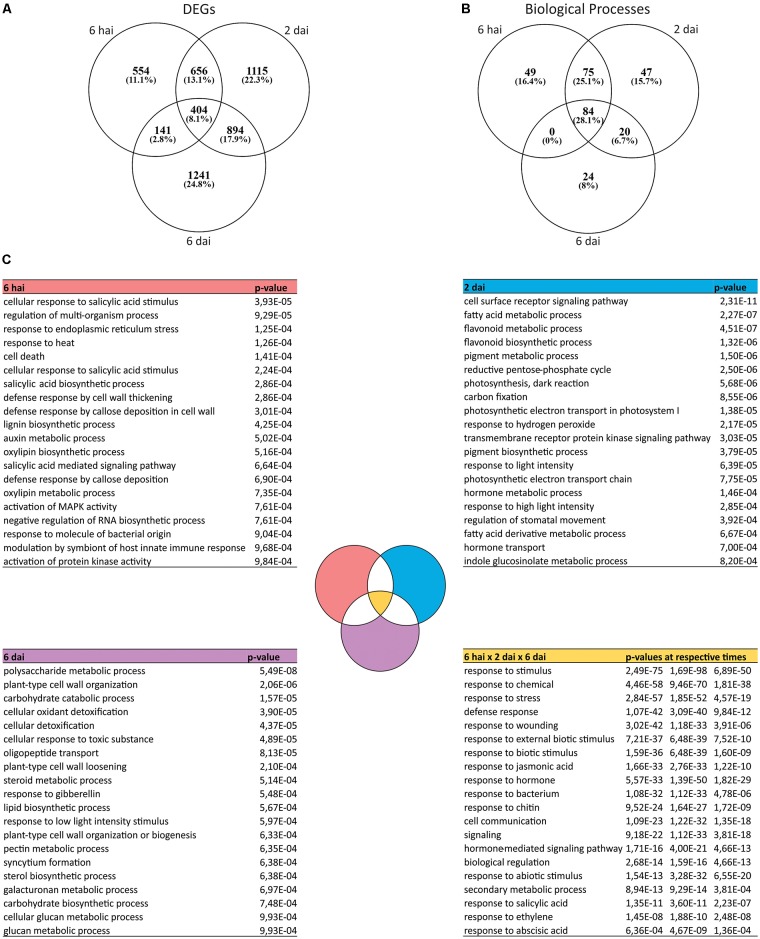
Transcriptomic changes at different time points after *Brevipalpus* mite infestation of *A. thaliana* plants. **(A)** Venn diagram of DEGs in mite-infested plants compared to non-infested control at each time point. DEGs were identified using *DESeq2* and defined by log_2_ fold-change ≥ 0.5 and FDR-corrected *p*-value ≤ 0.05. **(B)** Venn diagram of overrepresented BPs of DEGs at each time point. Overrepresented BPs were identified for each time point based on a hypergeometric test with FDR-adjusted *p*-values ≤ 0.001. **(C)** Lists of overrepresented BPs exclusive to each experimental time point (6 hai, 2 dai or 6 dai) and those commons between them (6 hai × 2 dai × 6 dai). BPs corresponding *p*-values obtained in the Gene ontology (GO) enrichment analysis are included in the right column of the tables. Twenty BPs of each list are presented in each table. Complete lists of exclusive and common BPs are available in **Supplementary Table S4**.

A comparison of the DEGs deregulated across the experiment revealed both common and specific changes at each time points (**Figure 2A**). Few DEGs were common to all time points, whereas the highest percentage of them were found to be exclusively modulated at 2 or 6 dai suggesting intense reprogramming steps of plant transcriptome throughout the course of the *Brevipalpus* mite infestation (**Figure 2A**).

Most of the BPs (84 terms) over-represented during mite infestation overlap at all time points (**Figure 2B** and **Supplementary Table S4**). These processes included most of the general terms of plant response to stresses and hormones, indicating a continuous and lasting reprogramming of the plant immune system since the beginning of the interaction until at least 6 dai. Several categories were common between 6 hai and 2 dai (75 terms), and 2 dai and 6 dai (20 terms), but no biological process was shared between the first and the last evaluated time points (**Figure 2B**).

Even though processes related to plant defense responses were markedly enriched over the time course of the experiment, time point-specific ontologies were also identified. From all the over-represented BP categories, 49, 47, and 24 were uniquely identified at 6 hai, 2 dai, and 6 dai, respectively (**Figure 2B** and **Supplementary Table S4**). Hormone biosynthesis (“salicylic acid biosynthetic process,” “oxylipin biosynthetic process”), early signaling (“activation of MAPK activity”), and structural defenses (“callose deposition,” “cell wall thickening,” and “lignin biosynthetic process”) were processes exclusively enriched at 6 hai (**Figure 2C**). At 2 dai, unique categories were related to the metabolism of defense-related secondary metabolites (“indole glucosinolate metabolic process,” “pigment,” and “flavonoid biosynthetic and metabolic process”), photosynthesis and oxidative stress (“photosynthesis,” “carbon fixation,” “photosynthetic electron transport,” “response to light intensity,” “response to oxidative stress”) (**Figure 2C**). Exclusive ontologies that came up with the late infestation state (6 dai) were detoxification processes (“cellular detoxification,” “cellular response to toxic substance”), and other associated to cell wall components and structure (“plant-type cell wall organization,” “cell wall loosening,” and “pectin,” “galacturonan,” “glucan,” “carbohydrate,” and “polysaccharide” metabolic process) (**Figure 2C**).

### *Brevipalpus* Mite Infestation Induces Plant Defensive Responses and Represses the Plant Growth-Related Processes

**FIGURE 3:**
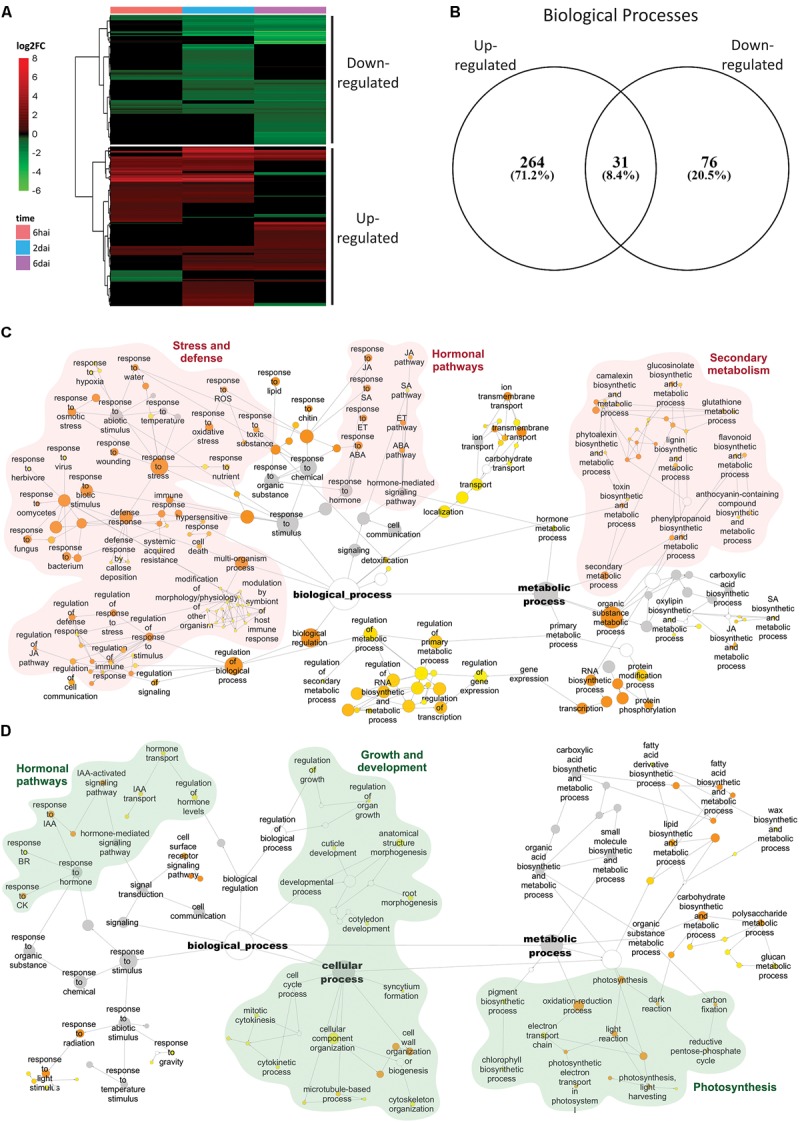
Induced and repressed responses on *A. thaliana* infested by *Brevipalpus* mites. **(A)** Hierarchical clustering analysis of log_2_ FC exhibited by DEGs on mite-infested plants compared to non-infested control. DEGs were identified using *DESeq2* and defined by log_2_ fold-change ≥ 0.5 and FDR-corrected *p*-value ≤ 0.05. hai, hours after infestation; dai, days after infestation. **(B)** Venn diagram of overrepresented BPs of DEGs at each cluster composed by up- and down-regulated genes. Overrepresented BPs were identified for each cluster based on a hypergeometric test with FDR-adjusted *p*-values ≤ 0.001. **(C,D)** Networks of enriched BPs from clusters of up-regulated **(C)** and down-regulated **(D)** DEGs, generated using the app BinGO in Cytoscape. Size of the nodes correlates with the number of DEGs. Color of the nodes reveals *p*-values of enriched categories. Nodes in gray represent categories that were shared between clusters of up- and down-regulated genes. Names of some BPs were simplified for clarity; full names are displayed in **Supplementary Table S5**. ROS, reactive oxygen species; SA, salicylic acid; JA, jasmonic acid; ET, ethylene; ABA, abscisic acid; IAA, auxin; CK, cytokinin; BR, brassinosteroid.

The vast majority of DEGs detected in more than one time point were strictly kept up- or down-regulated. Among the 5005 DEGs identified throughout the analysis, only 201 of them (4%) shift their expression patterns across the experiment (**Supplementary Table S2**). In agreement with this, results of the hierarchical clustering analysis revealed two major clusters of DEGs, which mainly encompassed 2762 and 2243 up-regulated and down-regulated genes, respectively (**Figure 3A**).

Gene ontology enrichment analysis separately performed with DEGs within each of the predefined clusters identified only 31 common categories between the up- and down-regulated groups (**Figure 3B** and **Supplementary Table S5**). These categories represented higher GO levels and included general BPs such as “regulation of biological quality,” “response to stimulus,” “metabolic process,” “signal transduction,” among others. BPs such as “response to hormones” and “hormone-mediated signaling pathway” were also shared between the up- and down-regulated clusters but GO-terms identifying a particular hormonal pathway were always detected in just one of the two groups.

The up-regulated cluster was enriched in 264 exclusive BPs (**Figure 3C** and **Supplementary Table S5**). Network topology was similar to that obtained using all the DEGs (**Supplementary Figure S2**), with two major clusters centralized in metabolic processes and response to stimuli. GO terms within metabolic process cluster involved several BPs related to secondary metabolism, whilst response to stimuli cluster presented terms associated to stress and defense and hormonal pathways. Over-represented categories were massively typified by defensive responses. Besides broad immune system-related terms (e.g., “immune response”), other common categories displayed by the general network (**Supplementary Figure S2**) included responses to hormones, oxidative stress and the production of secondary metabolites, e.g., glucosinolates, flavonoids, and camalexin. Only SA, JA, ET, and ABA-mediated hormonal pathways were represented in the up-regulated cluster network. Induced GO network also included other over-represented processes that were unidentified in the general network (**Supplementary Figure S2**). Among these are included: “response to herbivore,” “response to virus,” “multi-organism process,” “modification of morphology/physiology of other organism,” “lignin biosynthetic and metabolic process,” “defense response by callose deposition,” and “phytoalexin biosynthetic and metabolic process.”

The down-regulated cluster was enriched in 76 exclusive BPs, which were predominantly associated with the plant growth and development (**Figure 3D** and **Supplementary Table S5**). Over-represented terms included broad categories, for instance “developmental process” and “regulation of growth,” and also those directly related to plant growth such as “cytokinetic process,” “cell cycle process,” “mitotic cytokinesis,” or indirectly related to growth such as “cell wall organization or biogenesis” and “cytoskeleton organization”. Among the enriched BPs there were also processes associated to morphogenesis and development of specific plant components such as “root morphogenesis,” “cuticle development,” and “cotyledon development.” The other major class of over-represented BPs uniquely detected in the down-regulated cluster comprised photosynthesis-related processes such as “photosynthesis,” “electron transport chain,” “carbon fixation,” “photosynthesis, dark and light reaction,” “light harvesting,” “photosynthetic electron transport,” and “chlorophyll biosynthetic process.” Finally, the only hormones represented in the down-regulated cluster network were the major growth regulators IAA, CK and brassinosteroids (BR).

### Co-expression of Genes Correlates With Classes of Transcription Factors (TFs) Involved in SA, JA and Developmental Processes

Since transcriptional reprogramming is mainly controlled by TFs, the regulation of the expression dynamics of DEGs by specific classes of TFs was tested by two different approaches.

**FIGURE 4:**
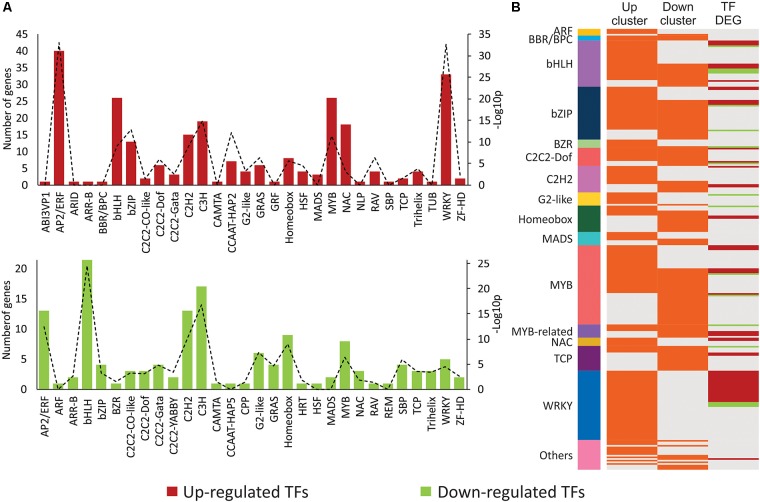
Enriched transcription factors (TFs) and TF targets in clusters of mite-responsive co-expressed genes. **(A)** Number of DEGs coding for TFs within each TF family identified in the clusters of up- and down-regulated DEGs. Up- and down-regulated DEGs are presented in red and green, respectively. Levels of enrichment (-Log_10_ *p*, with p: *p*-value) of each family (hypergeometric test, α ≤ 0.001) are presented by a dashed line with its corresponding values in the secondary axis. **(B)** TFs with enriched targets within each cluster of up- and down-regulated DEGs, identified by TF enrichment tool. TFs are grouped according to their families. Each line identifies one TF. In the first and second row (“Up” and “Down” clusters, respectively), orange lines correspond to TFs with enriched targets within each cluster. In the third row (“TF DEGs”), red and green lines represent up- and down-regulated differentially expressed genes, respectively, encoding TFs. Gray lines indicate absence of enriched targets for a given TF- and/or TF not differentially expressed. Families encompassing two or less TFs were grouped in “Others.”

First, over-represented TF families were searched based on up- and down-regulated DEGs that encode TFs (**Figure 4A** and **Supplementary Table S6**). Within the cluster of up-regulated DEGs, 254 (9.2%) TFs from 30 different families were identified. From those, 16 over-represented families were detected (hypergeometric test, α ≤ 0.001). The largest and most significant of them were the WRKY (33 genes, *p*-value = 2.47E-33) and the AP2/ERF (40 genes, *p*-value = 8.46E-34), known to act as regulators of SA pathway and ERF-branch of the JA pathway, respectively. From the analysis using the cluster of down-regulated DEGs, 141 (6.3%) TFs belonging to 30 families were detected. Twenty-three of these families were also found in the cluster of up-regulated DEGs. TFs were evenly distributed among 18 over-represented families (hypergeometric test, α ≤ 0.001), with lower significance (higher *p*-values). The largest and most significantly over-represented families were bHLH (22 genes, *p*-value = 3.21E-25), which comprises the regulators of the MYC-branch of the JA pathway, and C3H family (17 genes, *p*-value = 1.46E-17).

Second, TF families that potentially regulate the expression of the DEGs were searched based on over-represented target genes within the DEGs (**Figure 4B** and **Supplementary Table S7**). Enriched target genes and their corresponding TFs were identified by using a TF enrichment tool that takes advantage of previously identified *cis*-regulatory elements and regulatory interactions from literature mining (Jin et al., 2017). As a result, WRKY was the largest identified family with potential targets within the up-regulated DEGs. Twenty-one out of its 42 TF members were also induced during *Brevipalpus* mite-plant interactions. Targets from WRKY TFs were exclusively enriched in the up-regulated cluster. The next largest families with targets within the induced DEGs were MYB, bZIP, and bHLH, with 29, 26, and 24 TFs, respectively. Targets for MYB, bZIP, and bHLH, however, were not exclusively enriched in the analysis of the cluster of up-regulated DEGs. These families represented by 34, 24, and 14 TFs, respectively, were also among the largest with potential targets within down-regulated DEGs. MYC2, the marker TF from the MYC-branch of the JA pathway, was one of the bHLH TF with targets exclusively enriched in the down-regulated cluster. Notably, the analysis of the down-regulated cluster also revealed the TCP family, which is typically involved in the control of plant development. This family involved 15 and 4 TFs that potentially regulate targets within the assortment of repressed and induced DEGs, respectively.

Overall, the analysis of co-expressed genes with its corresponding TFs showed a correlation of up- and down-regulated genes with TFs that regulates SA, JA and developmental processes. The SA-related WRKY family was the largest one with target genes exclusively enriched in the up-regulated cluster and most of its TF members were also up-regulated. The analysis of enriched TFs settles the involvement of plant hormonal pathways and developmental processes in the plant response to *Brevipalpus* mites, with highlight on the participation of the SA pathway solely on the up-regulated responses.

### Focus on Defense Pathways: SA- and JA-Mediated Responses Are Induced in *Brevipalpus* Mite-Infested Plants

Over-represented genes from GO-terms associated with SA and JA-dependent responses were thoroughly reviewed to confirm their induced status. Data from genes included in the categories “response to SA” and “SA metabolic process,” or “response to JA,” “regulation of JA-mediated pathway,” and “JA metabolic process” were processed by a Hierarchical cluster analysis.

**FIGURE 5:**
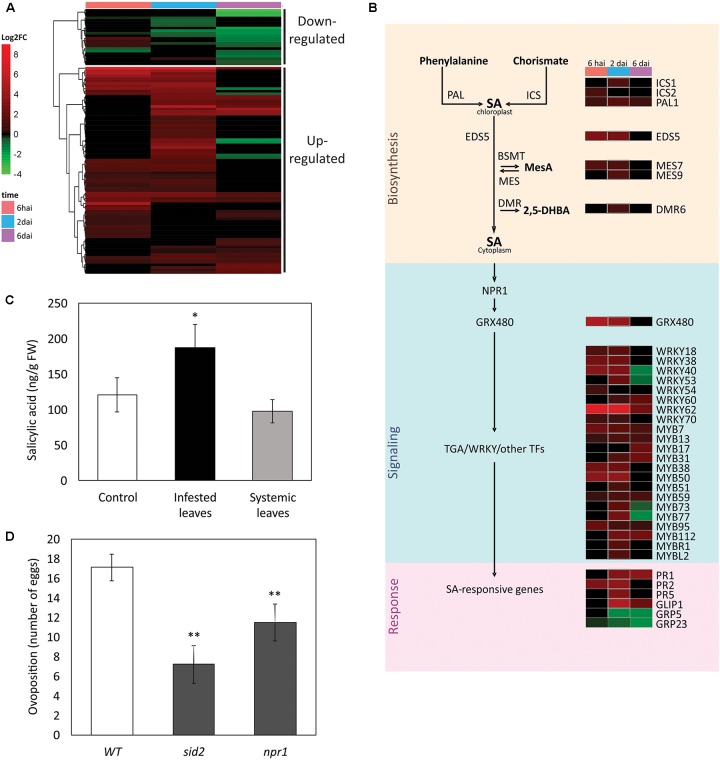
Salicylic acid (SA) pathway in the *A. thaliana* response to *Brevipalpus* mites. **(A)** Hierarchical clustering analysis of log_2_ FC exhibited by DEGs involved in the SA pathway. hai, hours after infestation; dai, days after infestation. **(B)** Schematic representation of SA pathway, where a sub-set of DEGs is presented. Colors identifies log_2_FC of DEGs at each experimental time point, according to the scale presented in **(A)**. **(C)** SA levels in infested and systemic leaves of mite-infested plants, and in non-infested control plants. Hormone levels were quantified by LC-MS/MS at 6 dai. Error bars represent standard errors. Statistically significant difference at *p*-value ≤ 0.05 (^∗^) is indicated. FW, fresh weight. **(D)** Mite performance in Arabidopsis mutants compromised in the SA pathway. Data represent the average number of eggs deposited after 4 days of infestation with five *Brevipalpus yothersi* mites/plant. Error bars represent standard errors. Statistically significant differences at *p*-values ≤ 0.01 (^∗∗^) are indicated. WT, wild type.

The SA-dependent pathway was represented by 103 DEGs (**Supplementary Table S8**). Eighty-one of these DEGs were up-regulated in at least one of the experimental time points. Some of these genes were induced at either early or late stages of the response, but they were not down-regulated in any of the other analyzed time points (**Figures 5A,B**). Examples of these expression patterns are the genes coding for the signaling protein for SA activation *EDS1* (*enhanced disease susceptibility 5*) and the SA-biosynthetic enzyme *ICS1* (*isochorismate synthase 1*) that were up-regulated at the beginning of the interaction, whilst the SA-responsive proteins *PR1* (*pathogenesis-related protein 1*) and *GLIP1* were induced at later time points. Moreover, the expression profile analysis of some DEGs revealed the quick regulation of some SA-responsive genes since the initial steps of the plant-mite interaction. For instances, *PR2/BGL2* (*pathogenesis-related protein 2/beta-1,3-glucanase 2*) was up-regulated as soon as 6 hai and remained activated at least till 2 dai. Among the induced genes associated to the SA pathway there were also several signaling kinases such as the receptor-like kinase (RLK) *CRK9* (*cysteine-rich RLK 9*), the *wall-associated kinases WAK1* and *WAKL10*, and the *L-type lectin receptor kinase LCRK-S.2, LCRK-IV.1* and *LCRK-IX.2.* Other up-regulated DEGs from this cluster were genes encoding the SA biosynthetic enzymes *ICS2* and *PAL1* (*Phenyl ammonia lyase 1*), the transporter of SA from chloroplast to cytoplasm *EDS5* (*enhanced disease susceptibility 5*), the regulator of SA responses *GRX480* (*glutaredoxin 480*), the methyl-salicylate (MeSA) esterase proteins *MES7* and *MES9* (*methyl esterase 7* and *9*), the defense protein *PR5*, and several TFS from WRKY and MYB families.

Another small cluster of SA-related genes comprised a group of 22 DEGs that were mainly down-regulated, particularly at the two latest time points of the experiment (**Figure 5A**). This cluster was largely formed by TFs that are also responsive to JA. Repressed TFs included members of the ERF/AP2, MYB and GRAS/DELLA families. Other repressed genes beyond TFs were *UGT1* (*UDP-glucose transferase 1*), involved in the callose formation, and the genes encoding the *GRP23* and *GRP5* proteins *(glycine-rich proteins 23 and 5*), which are components of the plant cell wall.

**FIGURE 6:**
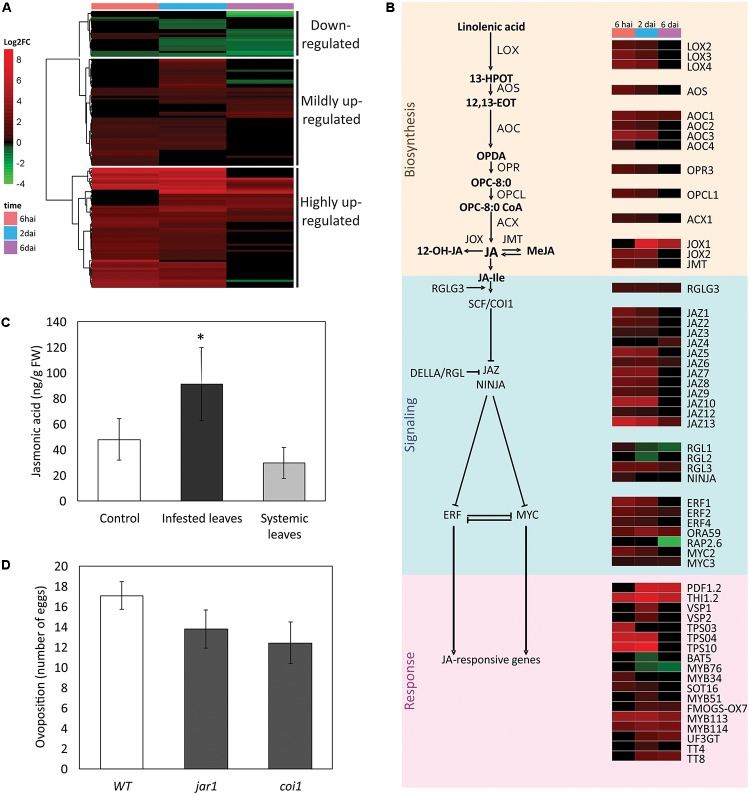
Jasmonic acid (JA) pathway in the *A. thaliana* response to *Brevipalpus* mites. **(A)** Hierarchical clustering analysis of log_2_ FC exhibited by DEGs involved in the JA pathway. hai, hours after infestation; dai, days after infestation. **(B)** Schematic representation of JA pathway showing a sub-set of DEGs. Colors identifies log_2_FC of DEGs at each time point, according to the scale presented in **(A)**. **(C)** JA levels in infested and systemic leaves of mite-infested plants, and in non-infested control plants. Hormone levels were quantified by LC-MS/MS at 6 dai. Error bars represent standard errors. Statistically significant differences at *p*-value ≤ 0.05 (^∗^) is indicated. FW, fresh weight. **(D)** Mite performance in Arabidopsis mutants compromised in the JA pathway. Data represent the average number of eggs deposited after 4 days of infestation with five *Brevipalpus yothersi* mites/plant. Error bars represent standard errors. WT, wild type.

The JA-mediated pathway was composed by 137 DEGs that, similarly to what was observed in the SA-pathway analysis, were mainly up-regulated (**Supplementary Table S8**) (**Figures 6A,B**). DEGs were subdivided in three clusters: two larger groups formed by 60 and 54 highly and mildly up-regulated genes, respectively, and a small one composed by 23 genes that were mostly down-regulated (**Figure 6A**).

Highly induced JA-related genes (**Supplementary Table S8**) at the beginning of the infestation code for proteins acting upstream on the JA pathway such as the DAMP receptor *PEPR2* (*PEP1 receptor 2*) and the JA-biosynthetic and modifying enzymes *AOS* (*allene oxide synthase*)*, AOC2* and *AOC3* (*allene oxide cyclase 2* and *3*)*, LOX2, LOX3,* and *LOX4* (*lipoxygenase 2*, *3,* and *4*), *OPCL1* (*OPC-8:0 CoA ligase 1*) and *JMT* (*JA carboxyl methyltransferase*) (**Supplementary Table S8**). DEGs induced at the two first experimental time points also included terpene synthases (*TPS03*, *TPS04,* and *TPS10*), several JAZ ( *jasmonate-zim-domain*) proteins (*JAZ1, JAZ2, JAZ5, JAZ7, JAZ8, JAZ9/TIFY7,* and *JAZ10*) and the TFs *MYC2* and *ERF1*. Genes with high expression at later time points encode proteins directly involved in defense such as the marker responsive protein of the ERF-branch *PDF1.2* (*plant defensin 1.2*), and proteins involved in the anthocyanin biosynthesis such as *UF3GT* (*UDP-glucose:flavonoid 3-o-glucosyltransferase*). Other highly up-regulated DEGs included MYB TFs, the DELLA protein *RGL3* that contributes to JA/ET-mediated defenses, the ERF-branch TFs *ERF2* and *ORA59* (*octadecanoid-responsive Arabidopsis AP2/ERF59*), the responsive gene *THI2.1* (*thionin 2.1*), and the JA oxidases *JOX1* and *JOX2* that down-regulate plant immunity by hydroxylation and inactivation of JA.

Genes from the JA pathway that were mildly induced (**Supplementary Table S8**) at the first time points included those coding for the JA-biosynthetic enzymes *ACX1* (*acyl-CoA oxidase 1*) and *AOC4*, the TF *WRKY70* that acts on the SA-JA antagonism, the negative regulators of JA-responsive genes *NINJA* (*novel interactor of JAZ*) and *JAZ3*, and the proteins *MYB34* and *SOT16* (*sulfotransferase 16*) involved in the synthesis of glucosinolates. Later in the infestation, the group of mildly induced genes included those coding for the proteins *MYB75/PAP1* and *TT4* that act in the biosynthetic pathway of anthocyanin, the *MYB51* and *FMOGS-OX7* proteins involved in the synthesis of glucosinolates, and the responsive marker genes of the MYC-branch *VSP1* and *VSP2* (*vegetative storage proteins 1* and *2*), which directly act during the anti-herbivory defense. Two essential modulator genes of the JA signaling, i.e., *RGLG3* (ring domain ligase 3) and the *MYC3* TF were induced at all the assessed time points although at low expression levels. *RGLG3* encodes for a RING-ubiquitin ligase acting upstream of JA-Ile recognition and MYC3 operates together with MYC2 coordinating the expression of responsive genes from the MYC-branch.

The down-regulated JA cluster comprised DEGs that were mainly repressed at 2 and 6 dai (**Supplementary Table S8**). Some of them encode TF commonly acting in the SA-pathway such as members of the families: ERF (*RAP2.6* and *DREB26*), MYB (*MYBS1*, *MYB28*, *MYB29*, and *MYB16*), and GRAS/DELLA (*RGL1* and *RGL2*). Other repressed genes were those encoding the *BAT5* (*bile acid transporter 5*) protein and the *MYB76* TF, both required for the biosynthesis of glucosinolates, the DAMP receptor *PEPR1*, and the JA-repressed protein *AGP31* (arabinogalactan protein 31).

### SA and JA Levels Increase in Mite-Infested Plants

Both SA and JA biosynthetic and responsive genes were induced in *Brevipalpus* mite-infested plants. To confirm the consistency of the observed molecular data, the profiles of the SA and JA hormones were determined in Arabidopsis plants challenged with *Brevipalpus* mites. SA (**Figure 5C**) and JA (**Figure 6C**) levels were 1.5- and 2.8-fold higher, respectively, on infested leaves when compared to the control ones (Student’s *t*-test, α ≤ 0.05).

Salicylic acid and JA levels were also verified in systemic leaves of mite-infested plants. No difference was observed between the levels of both hormones in systemic and non-infested control leaves, suggesting a local rather than a systemic response to *Brevipalpus* mite infestation.

### *Brevipalpus* Mites Have a Decreased Performance on Plants Impaired in SA Responses

Salicylic acid- and JA-mediated pathways were clearly induced upon *Brevipalpus* mite infestation. To further explain the functional relevance of the plant hormonal pathways on the interaction, the performance of *B. yothersi* mites was evaluated on Arabidopsis plants impaired in SA or JA-mediated response. Mite oviposition was assessed on mutants defective in SA biosynthesis (*salicylic acid induction deficient2, sid2*) and signaling (*non-expressor of pathogenesis-related protein1, npr1*), and JA signaling (*jasmonate resistant1, jar1* and *coronatine-insensitive1, coi1*). Plants were infested with adult female mites and the number of laid eggs was counted after 6 days.

The number of eggs per plant was 2.4- and 1.5-fold lower on SA-mutants *sid2* and *npr1*, respectively, when compared to the mite’s performance in the infested wild-type *A. thaliana* Col-0 plants used as control (Student’s *t*-test, α ≤ 0.05) (**Figure 5D**). No difference was observed between the number of eggs on the mutants affected in the JA pathway (*jar1* and *coi1*) and the wild-type control (Student’s *t*-test, α ≤ 0.05) (**Figure 6D**). These results point to a role of SA-mediated response promoting the *Brevipalpus* mite colonization.

## Discussion

False-spider mites of the genus *Brevipalpus* are serious and cosmopolite phytophagous pests with a unique biology (Weeks et al., 2001). They directly provoke injuries in some plant species, but the major consequence of their feeding behavior ensues from the transmission of several cile- and dichorha- viruses that infect economically important crops (Kitajima and Alberti, 2014). Almost 10 species of *Brevipalpus* mites are known to act as virus vector, but, among them, mites of the species *B. yothersi* stands out due to their involvement in transmission of viruses causing citrus leprosis, a severe disease that threatens the citrus industry in the Americas (Beard et al., 2015; Ramos-González et al., 2016). To disentangle the *Brevipalpus*-mite interaction, in the current paper we provide data that extensively describe the response of Arabidopsis plants during their colonization by *Brevipalpus* mites. Changes in the plant transcriptome profile are complemented with the analysis of the accumulation of defense hormones and the results are discussed emphasizing the role of particular plant defense genes during the *Brevipalpus* infestation process.

Our results showed that mite infestation clearly triggers the plant immune system. Processes related to the response to herbivory and other biotic stresses dominate a large number of the over-represented GO categories among all DEGs. Most of the BPs were common between all the time points, although specific changes were also identified. Plant response during the initial 6 h included the induction of genes involved in the hormone biosynthesis and signaling, consistent with an early recognition of the mite feeding. Transcriptome changes were followed by the up-regulation of a wide range of genes involved in defense and synthesis of secondary metabolites at 2 and 6 dai. The major dissimilarity was between the first and last time points, which do not share any BPs except the ones that were present throughout the infestation. GO enrichment analysis revealed that defense responses were up-regulated and mainly involved the SA- and JA-mediated pathways. Deeper analysis on SA/JA-related DEGs and quantification of hormone contents confirmed that these pathways were distinctly induced upon the infestation by *Brevipalpus* mites. On mite-infested plants, genes involved in the biosynthesis, signaling, and response of the SA and JA pathways were mostly up-regulated, and SA and JA hormone levels were increased. In this regard, simultaneous induction of SA and JA plant response to *B. yothersi* infestation follows a similar pattern to those observed during plant colonization by several spider mites (Kant et al., 2004; Zhurov et al., 2014; Alba et al., 2015; Rioja et al., 2017). Induced defense-related processes also included a clear transcriptional response to oxidative stress. Previous histochemical analysis of infested tissues revealed the production of ROS upon mite feeding (Arena et al., 2016). Induction of ROS production and related transcripts also resembled plant response to spider mite feeding and, in both cases, the role of ROS signaling remains to be determined (Agut et al., 2018).

Gene ontology enrichment analysis revealed an extensive genetic expression adjustment throughout the JA pathway including the hormonal biosynthesis and metabolism, and downstream regulation and response. Mite presence induced the DAMP-receptor PEPR2, suggesting the perception capacity of damaged tissues by Arabidopsis plants. Although minimal, mite feeding causes tissue disruption on infested leaves (Arena et al., 2016). Individual or very few dead cells are observed after mite feeding activity, probably as consequence of punctures by the mite stylets. Upon recognition, JA-biosynthetic enzymes such as AOC, AOSs, and LOXs were up-regulated. Higher JA content in mite-infested leaves confirmed activity of this biosynthetic pathway. Downstream of JA production, several signaling proteins and regulators were induced, including many TFs from MYB, AP2/ERF, and bHLH families. Downstream responses were represented by an array of up-regulated transcripts involved in the production of terpenes, anthocyanin, and glucosinolates. Since induced JA responses to *Brevipalpus* mites are similar to the ones that mediate Arabidopsis response to spider mites (Zhurov et al., 2014), our results indicate a conservation of mite-induced JA regulatory mechanisms. Moreover, several negative regulators of JA response were induced on plants infested by *B. yothersi*, including genes encoding NINJA and numerous JAZ proteins, which interact to repress the TFs that regulates the expression of JA-responsive genes (Wager and Browse, 2012), and JA oxidases, which down-regulates downstream responses by hydroxylation and inactivation of JA (Caarls et al., 2017). In this context, the induced JA pathway might be attenuated, and consequently, the observed data reflect a somewhat mitigated rather than a fully-induced JA-mediated response.

Even though the JA pathway was largely induced upon mite infestation, distinct activation profiles of JA branches were observed. First, TFs from the ERF- and MYC-branches were differentially regulated. AP2/ERF family with TFs that control the ERF-branch was the largest and most enriched family within up-regulated DEGs, whilst bHLH family that includes the TFs that regulates the MYC-branch was the largest and most enriched one within down-regulated DEGs. Particularly, MYC2 that is the major regulator of the MYC-branch responsive genes, was induced, although its target genes were enriched within the cluster of down-regulated genes. Second, the expression levels of defensive genes from the ERF-branch were much higher than that from genes of the MYC-branch. The gene encoding the ERF-responsive anti-microbial protein PDF1.2 figures among the most highly up-regulated DEGs (e.g., FC = 94-fold at 2 dai), whilst those coding for the MYC-responsive anti-herbivory proteins VSP2 and VSP1 were only mildly or not induced at the same experimental time points (e.g., FC = 4- and 10-fold, respectively, at 2 dai). The preferential activation of the ERF-branch over the MYC-branch was described as an herbivore strategy to induce a harmless response in expense of a harmful defense (Verhage et al., 2011; Pieterse et al., 2012). The strongest activation of the ERF-branch reported here corroborates a previous study proposing that *Brevipalpus* mites might mitigate effective defenses by manipulating the plant resistance mechanisms toward herbivore preferred JA responses (Arena et al., 2016). However, further analysis of ERF and MYC mutants are required to clarify the actual role of each one the JA branches in plant response to *Brevipalpus* mites.

Within the induced hormonal pathways in response to *Brevipalpus* infestation, the SA-mediated pathway plays a conspicuous role. On these plants SA levels were elevated, the vast majority of SA-related genes were up-regulated, and the SA-related WRKY TFs as well as their target genes were exclusively over-represented in the cluster of up-regulated genes. Induction of SA response has been associated with stealthy arthropods such as piercing-sucking insects (Nguyen et al., 2016; Patton et al., 2017). Likewise, *Brevipalpus* mite feeding behavior causes minimal tissue disruption. During feeding, mites pierce epidermal cells using interlocked stylets, sometimes through leaf stomata, and suck out overflowed cell content (Kitajima and Alberti, 2014). Activation of the SA pathway by *Brevipalpus* mites agrees with the common pattern observed for herbivores causing little overt tissue damage (Arimura et al., 2011).

Interestingly, an increasing number of evidence indicate that activation of SA pathway favors herbivore performance rather than acts as an effective defense against herbivory. For instance, *Bemisia tabaci* nymphs performs better in the *cpr6* mutants pre-activated for SA-mediated defenses (Zhang et al., 2013), and SA exogenous application render Arabidopsis plants more attractive to thrips (Abe et al., 2012). Using Arabidopsis mutants, we found that the performance of *Brevipalpus* mites is compromised in plants with lower SA content (*sid2*, mutant for ICS1) and defective SA signaling (*npr1*). In comparison with wild-type plants, the number of laid eggs was 2.4- and 1.5-fold lower on *sid2* and *npr1* mutant plants, respectively. Whilst SA levels during plant-biotic stresses is mainly produced through ICS1-mediated isochorismate pathway (Wildermuth et al., 2001), responses downstream SA accumulation are branched in NPR1-dependent and -independent genes (Uquillas et al., 2004; Shearer et al., 2012). The milder phenotype from *npr1* in comparison with *sid2* might be related to intact NPR1-independent responses. Influence of SA response in *Brevipalpus* mites seems contrary to its role against spider mites. Even though a few reports showed no influence of SA against *Tetranychus* species (Zhurov et al., 2014), a recent study showed that *T. urticae* mites have an increased performance on SA-deficient NahG tomato plants (Villarroel et al., 2016). Lower oviposition of *B. yothersi* mites in either the SA -synthesis or -signaling impaired plants suggests the manipulation by *Brevipalpus* mites of the SA pathway aiming the promotion of host colonization. The positive influence of the SA pathway over *B. yothersi* also has implications for the role of the mite as a vector. We previously reported that infestation with CiLV-C-carrying *B. yothersi* induces even stronger SA response than that reached during non-viruliferous mites feeding (Arena et al., 2016). Higher up-regulation of SA pathway in response to viral infection might further enhance mite colonization and, probably, contribute to the viral transmission.

Salicylic acid-mediated improvement of herbivore performance is usually associated with the antagonistic interactions between the SA and JA signaling pathways (Bruessow et al., 2010; Thaler et al., 2012; Zhang et al., 2013; Caarls et al., 2015). Some arthropods induce SA as a strategy to repress JA effective defenses exploiting the natural cross-talk between signaling pathways. JA defenses, and specifically the production of indole glucosinolates, are central to Arabidopsis defense against *Tetranychus* species (Rioja et al., 2017). Higher reproduction rate of *Brevipalpus* mite due to the induction of the SA pathway could be associated with the reduction on the JA pathway, as previously suggested (Arena et al., 2016). However, our current results show that *Brevipalpus* mite oviposition was not increased in Arabidopsis mutants impaired in JA-responses (*jar1* and *coi1*), therefore, the role of JA pathway against *Brevipalpus* mite colonization is not as obvious as against spider mites, or it does not directly affect oviposition. Molecularly, our results might suggest that the induction of SA antagonizes a set of JA responses which are independent of JAR1 and COI1, or that the SA pathway might improve mite performance by mechanisms alternative to the SA–JA crosstalk. It is noteworthy that upon mite infestation both *JAR1* and *COI1* genes were not induced (**Supplementary Table S2**), consequently, at least at transcriptional level, evidence of involvement of these two gene in response against *Brevipalpus* mite colonization was not revealed. Furthermore, it is possible that the JA pathway influences other aspects rather than mite oviposition such as host preference or mite development. The deeper analysis of other JA mutants and features of mite behavior will help to disentangle the role of the JA on mite infestation.

Some arthropod herbivores are capable of manipulating host responses to circumvent defenses (Stahl et al., 2018). Even though most of the known examples of defense suppression by herbivores involves plant–insect interactions, some cases of suppressive mites have been described (Agut et al., 2018). Defense counteraction has been shown to occur by secreted proteins, called effectors, injected into host cells through herbivores’ saliva to interfere with plant responses (Hogenhout and Bos, 2011). Effectors from *Tetranychus* saliva that suppress harmful defenses and increase spider mite performance were recently described (Villarroel et al., 2016). *Brevipalpus* mites likely inject saliva inside host cells through a tube formed between its interlocked stylets (Kitajima and Alberti, 2014). The ability of *B. yothersi* mites to manage the plant response favoring their own performance suggests that, similarly as spider mites do, *Brevipalpus* mites might also deliver saliva-borne effector proteins into plant cells. It is noteworthy, however, that mites from *Brevipalpus* genus employ such a distinct strategy of modulation of plant responses compared to closely-related spider mites. Even though feeding by both *Brevipalpus* and *Tetranychus* mites induce SA and JA pathways simultaneously, the effectiveness of such responses diverges between the two systems. Whilst JA pathway defend plants against *Tetranychus* mites (Zhurov et al., 2014), SA pathway has been described as neutral or detrimental to spider mite species (Villarroel et al., 2016). On the contrary, adverse effect of JA responses to counteract *Brevipalpus* mite infestation was not revealed in the present study, but SA pathway has a positive effect over the colonization of these false-spider mites, pointing to a unique response within described plant-mite interactions.

Polyphagous arthropods likely posses a larger collection of salivary proteins due to their exposure to a wide range of host plants with distinct morphology and physiology (Vandermoten et al., 2014). Large groups of proteins families identified in *T. urticae* saliva were proposed to facilitate the expansion of the host range of these highly polyphagous mites (Jonckheere et al., 2016). Like *T. urticae*, *Brevipalpus* mites infest a wide range of hosts that includes almost a thousand of plant species in more than a hundred of different families (Childers et al., 2003). Collectively, our results suggest that *Brevipalpus* mites manipulate the plant defensive response to render the plant more susceptible to the colonization by inducing the SA-mediated pathway, a mechanism unusual to spider mite species. Mite’s ability to modulate the plant physiology in their favor might support the high polyphagous nature of false-spider mites.

Independent GO enrichment analysis from up- and down-regulated DEGs revealed not only the up-regulation of defensive responses, but also the repression of plant growth-related processes. Defensive responses have been long considered to impose a cost that results in reduced plant growth and reproduction (Züst and Agrawal, 2017). Growth-defense trade-off comes from a reallocation of resources to optimize fitness when plants are exposed to environmental changes. Upon herbivory, the plant metabolism is frequently reconfigured. While the secondary metabolism is enhanced to produce defenses, the primary metabolism is suppressed. For instance, induction of JA pathway by *Manduca sexta* results in down-regulation of photosynthesis in *Nicotiana attenuata* (Halitschke et al., 2011). Plant growth genes repressed during the elicitation of defenses comprise, among others, those associated with cell wall (e.g., expansins), cell division (e.g., cyclins), and DNA replication and photosynthesis (such as components of the light-harvesting complex, photosystem subunits, electron transport chain, chlorophyll biosynthesis, etc.) (Attaran et al., 2014). On *Brevipalpus* mite-infested plants, several of those growth-related genes were down-regulated. Over-represented GO terms within repressed DEGs included processes associated to the cell wall, morphogenesis of cell components, cell division, and photosynthesis.

The plant growth-defense trade-off is modulated through the interplay between defense hormonal pathways mediated by SA and JA and the hormones that act as the major plant growth regulators, i.e., IAA, BR, and GA. Some molecular players that regulate the trade-off have been identified (Huot et al., 2014; Lozano-Durán and Zipfel, 2015; Campos et al., 2016; Züst and Agrawal, 2017). DELLA proteins are key negative regulators of GA signaling that inactivates growth-promoting phytochrome-interacting factors (PIFs). Upon GA elicitation, DELLA proteins are degraded, releasing PIFs and allowing them to activate expression of growth-promoting genes (Huot et al., 2014; Züst and Agrawal, 2017). DELLA and JAZ proteins interact, inhibiting each other actions over the repression of growth and defense-related genes (Yang et al., 2012). The degradation of JAZ proteins triggered by JA accumulation releases DELLA from JAZ binding, thereby strengthens the suppression of PIFs and plant growth (Yang et al., 2012; Züst and Agrawal, 2017). Likewise, overexpression of the DELLA protein RGL3 reduces GA-mediated growth while increases MYC2-dependent expression of JA-responsive genes (Wild et al., 2012). Our results indicate that markers from the molecular mechanism behind the trade-off, such as RGL3, were induced. The SA- and JA-dependent defense responses were up-regulated and IAA-, BR-, and GA-mediated growth processes were downregulated, suggesting that the growth-defense trade-off occurs during Arabidopsis-*Brevipalpus* interaction.

Results obtained here extend our previously proposed model on the Arabidopsis response to non-viruliferous *Brevipalpus* mites (Arena et al., 2016). Beyond the responses focused here, the large-scale transcriptome we obtained will provide a valuable resource to further explore unknown molecular components involved in plant interaction with false-spider mites.

## Materials and Methods

### Plant Material

Wild-type A. thaliana ecotype Columbia (Col-0) was obtained from the Arabidopsis Biological Resource Center^1^. Arabidopsis mutants in the Col-0 background (sid2-1, npr1, and jar1) were obtained from Georg Jander. The Arabidopsis mutant coi1-16 was obtained from Kirk Overmyer. Plants were grown in controlled growth chambers (Conviron, Winnipeg, Canada) at 23 ± 2°C and a 12 h light/dark photoperiod. Four-week-old plants were used in the experiments.

### Mite Rearing

Non-viruliferous mites were initially obtained from citrus orchards and further confirmed as *B. yothersi* using phase contrast microscopy as reported elsewhere (Beard et al., 2015). Mites were reared onto fruits of ‘Tahiti’ acid lime (*Citrus latifolia* Tanaka), a genotype immune to citrus leprosis virus C, as previously described (Arena et al., 2016). Mites were reared for several generations and were confirmed as non-viruliferous by RT-PCR using primers for CiLV-C (Locali et al., 2003) before their use in the experiments.

### RNA-Seq Experiment

A time course experiment was conducted on plants infested with non-viruliferous mites and on non-infested control plants at 6 h after infestation (hai), 2 and 6 dai. For each time point, Arabidopsis Col-0 plants were grouped in sets of 16 individuals assigned to each treatment (infested and control). Plants from both treatments were kept at the same growth chamber. Plants from the infested treatment were challenged with 15 mites (5 mites per each of 3 rosette leaves), transferred with a small brush under a stereoscopic microscope. Mites were not caged. Infested or control leaves were collected at each time-point. From mite-infested plants, only the leaves where mites were originally deposited were collected. Leaves from two plants were pooled, totaling eight biological replicates per treatment per time point, flash-frozen in liquid N_2_ and stored at -80°C until RNA extraction. Plant RNA was purified using the RNeasy Plant Mini Kit (Qiagen, Venlo, Netherlands) and treated RNAse-free DNAse (Qiagen, Venlo, Netherlands) for removal of residual plant DNA. RNA quality was assessed in Bioanalyzer 2100 (Agilent technologies, Santa Clara, CA, United States). All samples had an RNA integrity number (RIN) above eight and were considered suitable for RNA-Seq. RNA extracts from two samples (100 ng/μL each) were pooled in a single sample, totaling four replicates per treatment per time point for library construction and independent sequencing. cDNA libraries were prepared using Illumina TruSeq Stranded mRNA Library Prep Kit (Illumina, San Diego, CA, United States). Sequencing was performed with HiSeq SBS v4 High Output Kit (Illumina, San Diego, CA, United States) in an Illumina HiSeq 2500 system (Illumina, San Diego, CA, United States) and generated 2 × 125 bp paired-end reads.

### Bioinformatics Analysis of RNA-Seq Data

RNA-Seq data were analyzed using R and Bioconductor according to Anders et al. (2013) with some modifications. Quality of the sequences was confirmed using ShortRead (Morgan et al., 2009) and FASTQC. Reads were mapped to the *A. thaliana* TAIR10 genome using TopHat2 (Kim et al., 2013). The number of reads per gene was counted with HTSeq (Anders et al., 2015) and normalized by size factors obtained from the negative binomial-based DESeq2 package (Love et al., 2014). After normalization, clusterization profiles of the samples were assessed by hierarchical clustering (with Euclidean distance metric and Ward’s clustering method) and principal component analysis (PCA). Differentially expressed genes (DEGs) between infested and control treatments were identified at each time point using likelihood ratio tests after negative binominal fittings using the package DESeq2 (Love et al., 2014). Genes with False Discovery Rate (FDR)-corrected *p*-values ≤ 0.05 and fold-change (log_2_) threshold of 0.5 were classified as differentially expressed. To identify mechanisms potentially involved in the plant response to mite feeding, GO Enrichment Analysis was performed. A gene set was defined as all DEGs (unless otherwise noted) and the universe comprised all genes of the *A. thaliana* TAIR10 genome expressed in at least one of the observed conditions. Overrepresented BPs, MFs, and CCs were identified based on a hypergeometric test with FDR-adjusted *p*-values ≤ 0.001. GO networks were generated using the app BinGO in Cytoscape (Maere et al., 2005).

### Identification of Enriched Transcription Factors

A hierarchical clustering was performed with all DEGs to identify up- and down- regulated clusters, using Euclidean distance metric and Ward’s clustering method. Two approaches were used to identify enriched TFs on each cluster. First, we searched for genes coding for TFs within DEGs using PlantTFDB database (Jin et al., 2017). Over-represented TFs on each cluster were identified using a hypergeometric test (α ≤ 0.001). Second, we searched for enriched TF targets using the TF enrichment tool, based on previously identified *cis*-regulatory elements and regulatory interactions from literature mining (Jin et al., 2017).

### Validation of Gene Expression Data by RT-qPCR

Another time course experiment was set with plant infested with non-viruliferous mites and non-infested control plants at 6 hai, 2 and 6 dai. For each time point, Arabidopsis Col-0 plants were grouped in sets of 16 individuals assigned to each treatment (infested and control). Plants from the infested treatment were challenged with 15 mites (5 mites per each of 3 rosette leaves). Infested or control leaves were collected at each time-point. Leaves from two plants were pooled, totaling eight biological replicates per treatment per time point, and flash-frozen in liquid N_2_. Plant RNA was purified using the RNeasy Plant Mini Kit (Qiagen, Venlo, Netherlands) and treated with RNAse-free DNAse (Qiagen, Venlo, Netherlands). RNA concentration was assessed using NanoDrop ND-8000 microspectrophotometer (Thermo Scientific, Waltham, MA, United States) and RNA quality was verified in 1.2% agarose gels. cDNA were generated for each RNA sample (500 ng) using RevertAid H Minus First Strand cDNA Synthesis Kit (Thermo Scientific, Waltham, MA, United States) as described by the manufacturer. RT-qPCR were prepared with 6.5 μL of GoTaq qPCR Master Mix (Promega, Madison, WI, United States), 120 nM of each gene-specific primer pair and 3 ng of cDNA. Primer sequences are available on **Supplementary Table S9**. Reactions were performed in a 7500 Fast Real-Time PCR System (Thermo Scientific, Waltham, MA, United States) device, using the standard settings. Each sample was analyzed in triplicate and melting curves were included to confirm the absence of genomic DNA and unspecific reactions. Quantification cycle (*C*q) values and primer pairs efficiencies were determined for each individual reaction using Real-time PCR Miner (Zhao and Fernald, 2005). Gene expression analyses were performed according the Δ*C*q model using multiple reference genes (Hellemans et al., 2007) as previously described (Arena et al., 2016). Statistical significances between infested and control samples within each time point were assessed using Student’s *t*-test (α ≤ 0.05).

### Quantification of Hormone Levels

Four-week-old Arabidopsis Col-0 plants were infested with 10 mites (two leaves with five mites each) or kept without mites. Infested leaves were collected after 6 days. Leaves from two plants were pooled together in one sample, totaling six replicates per treatment. Harvested leaves were weighted, flash frozen in liquid nitrogen and ground in a paint shaker. The SA and JA contents at local and systemic leaves of mite-infested plants were compared with those from the non-infested control as previously described (Casteel et al., 2015). For analysis, 5 μL of each extract were analyzed on a triple-quadrupole liquid chromatography-tandem mass spectrometry system (Agilent 6420A triple-quadrupole with Infinity II HPLC). Extracts were separated on a Zorbax Extend-C18 HPLC column (Agilent, 3.5 μm, 150 mm × 3.00 mm) using 0.1% formic acid in water and 0.1% formic acid in acetonitrile. Statistical significance was assessed using Student’s *t*-test (α ≤ 0.05).

### Mite Performance in Arabidopsis

The mite performance was evaluated on Arabidopsis mutants impaired in SA- (*sid2* and *npr1*) or JA- (*jar1* and *coi1*) mediated response. Plants were infested with five female adult *B. yothersi* mites in a single leaf, caged to prevent escape, and a completely randomized design was set. After 4 days of infestation, plant leaves were carefully detached, and the number of mite eggs was counted. Data from each mutant genotype was compared to the wild-type plants using Student’s *t*-test (α < 0.05).

### RNA-Seq Raw Data

The RNA-Seq raw data are available at sequence read archive (SRA) with the ID SRP144249.

## Author Contributions

GA, PR-G, CC, JF-A, and MM conceived and designed the experiments. GA, PR-G, and LR performed the experiments. GA, PR-G, and MR-A analyzed the data. MR-A, CC, MM, and JF-A contributed with reagents, materials, and analysis tools. GA, PR-G, and JF-A wrote the paper. All authors discussed the results and reviewed the final manuscript.

## Conflict of Interest Statement

The authors declare that the research was conducted in the absence of any commercial or financial relationships that could be construed as a potential conflict of interest.

## Footnotes

1 http://www.arabidopsis.org

## Supplementary Material

FIGURE 1 RT-qPCR validation of the RNA-Seq data. Expression profile of selected *Arabidopsis thaliana* genes was analyzed in *Brevipalpus yothersi* mite-infested plants by RNA-Seq and RT-qPCR. Data are presented as log_2_ fold-change (FC) values in comparison with non-infested plants (with log_2_FC set to zero). Statistically significant differences of mite-infested versus non-infested control at *p*-values ≤ 0.01 (^∗∗^) and ≤ 0.05 (^∗^) are indicated. hai, hours after infestation; dai, days after infestation. Note that the gene coding for the SA-responsive protein *PR1* was up-regulated at 2 and 6 dai, following the same profile obtained by RNA-Seq. The SA-related genes *GRX480*, *WRKY70*, and *PR2* were also identified as induced by RT-qPCR at the beginning of the interaction, but statistical significance was not confirmed in the later time points. Expression of the JA-responsive TFs *MYC2* and *ORA59* was also validated by RT-qPCR. Even though *MYC2* transcripts were significantly higher at 6 dai by RT-qPCR but not RNA-Seq, up-regulation of this TF at 6 hai and 2 dai was identified using both techniques. Likewise, *ORA59* gene was up-regulated at 6 hai and 6 dai by both RNA-Seq and RT-qPCR. RT-qPCR results also confirmed the mild induction of the MYC-branch responsive gene *VSP2* at 2 dai, and the high induction of the ERF-branch marker gene *PDF1.2* at 2 and 6 dai. The growth-related gene *EXP3* was down-regulated at 6 hai and 6 dai, while the negative regulator *RGL3* was induced at the same time points, according the RNA-Seq data.

FIGURE 2 Main biological processes (BPs) affected by *Brevipalpus* mites in *Arabidopsis thaliana* plants. Overrepresented BPs were identified based on a hypergeometric test with false discovery rate (FDR)-adjusted *p*-values ≤ 0.001. Gene ontology (GO) networks were generated using the app BinGO in Cytoscape. Color and size of the nodes identify the number and *p*-values of differentially expressed genes (DEGs) from each category. Names of some BPs were simplified for clarity; full names are shown on **Supplementary Table S3**. ROS, reactive oxygen species; SA, salicylic acid; JA, jasmonic acid; ET, ethylene; ABA, abscisic acid; IAA, auxin; CK, cytokinin; GA, gibberellic acid.

TABLE 1 Sequencing and alignment statistics for all *Arabidopsis thaliana* samples. hai, hours after infestation; dai, days after infestation.

TABLE 2 Total differentially expressed genes (DEGs) in *A. thaliana* plants infested with *Brevipalpus yothersi* mites at 6 hours after infestation (hai), 2 days after infestation (dai) and 6 dai. FC, fold-change; FDR, False Discovery Rate-corrected *p*-values. Gene symbols and descriptions were retrieved from ThaleMine (https://apps.araport.org/thalemine).

TABLE 3 Enriched Biological processes (BPs), molecular functions (MFs), and cellular components (CCs) in the set of DEGs of *A. thaliana* plants infested with *B. yothersi* mites. Overrepresented BPs, MFs, and CCs were identified based on a hypergeometric test with False Discovery Rate (FDR)-adjusted *p*-values ≤ 0.001. GO, Gene ontology; FDR, FDR-corrected *p*-values.

TABLE 4 Enriched BPs at each time point assessed after the infestation of *A. thaliana* plants with *B. yothersi* mites. Overrepresented BPs were identified based on a hypergeometric test with FDR-adjusted *p*-values ≤ 0.001. GO, Gene ontology; FDR, FDR-corrected *p*-values; hai, hours after the infestation; dai, days after the infestation.

TABLE 5 Enriched BPs at each cluster formed by DEGs that were mainly up- or down-regulated during the *A. thaliana* interaction with *B. yothersi* mites. Clusters were defined after a hierarchical clustering analysis of all DEGs. Overrepresented BPs were identified based on a hypergeometric test with FDR-adjusted *p*-values ≤ 0.001. GO, Gene ontology; FDR, FDR-corrected *p*-values.

TABLE 6 Differentially expressed genes (DEGs) coding for TF at each cluster formed by DEGs that were mainly up- or down-regulated during the *A. thaliana* interaction with *B. yothersi* mites. Clusters were defined after a hierarchical clustering analysis of all DEGs.

TABLE 7 Transcription factors (TFs) with enriched targets within each cluster formed by DEGs that were mainly up- or down-regulated during the *A. thaliana* interaction with *B. yothersi* mites. TFs with enriched targets were identified by TF enrichment tool (Jin et al., 2017). Up- and down-regulated clusters were defined after a hierarchical clustering analysis of all DEGs.

TABLE 8 Differentially expressed genes (DEGs) involved in salicylic acid (SA) and jasmonic acid (JA) pathways, identified in *A. thaliana* plants infested with *B. yothersi* mites at 6 hai, 2 dai and 6 dai. FC, fold-change; FDR, False Discovery Rate-corrected *p*-values. Gene symbols and descriptions were retrieved from ThaleMine (https://apps.araport.org/thalemine).

TABLE 9 Selected genes and corresponding primer pairs of *A. thaliana* used for gene expression analyses by RT-qPCR.
