# Supplementary figures and images for: Making a Better Home: Modulation of Plant Defensive Response by Brevipalpus Mites
Source: Front Plant Sci. 2018 Aug 15;9:1147. doi: 10.3389/fpls.2018.01147 (PMC6104575; doi:10.3389/fpls.2018.01147)

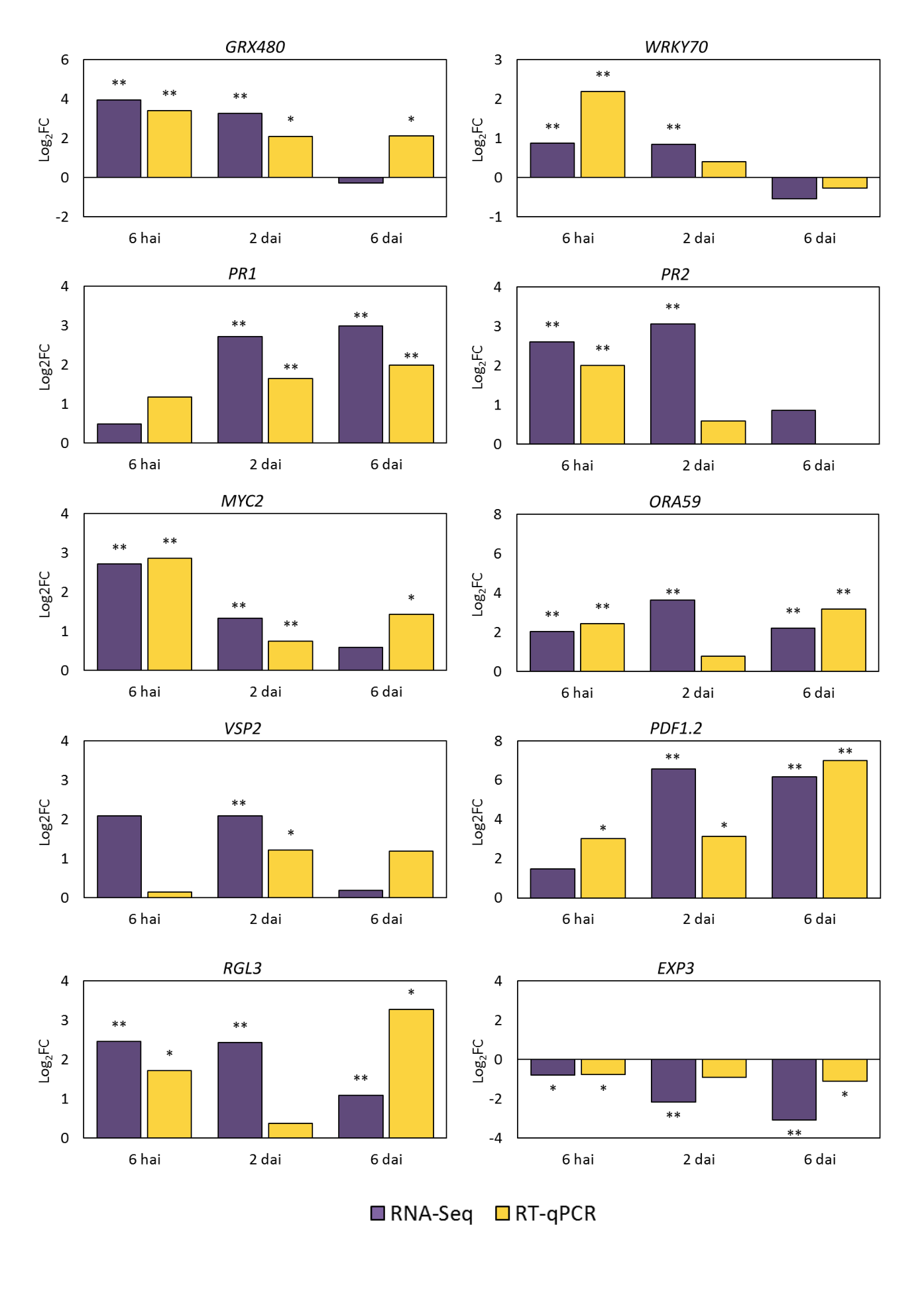

Supplement: FIGURE 1 [file Image_1.TIFF]

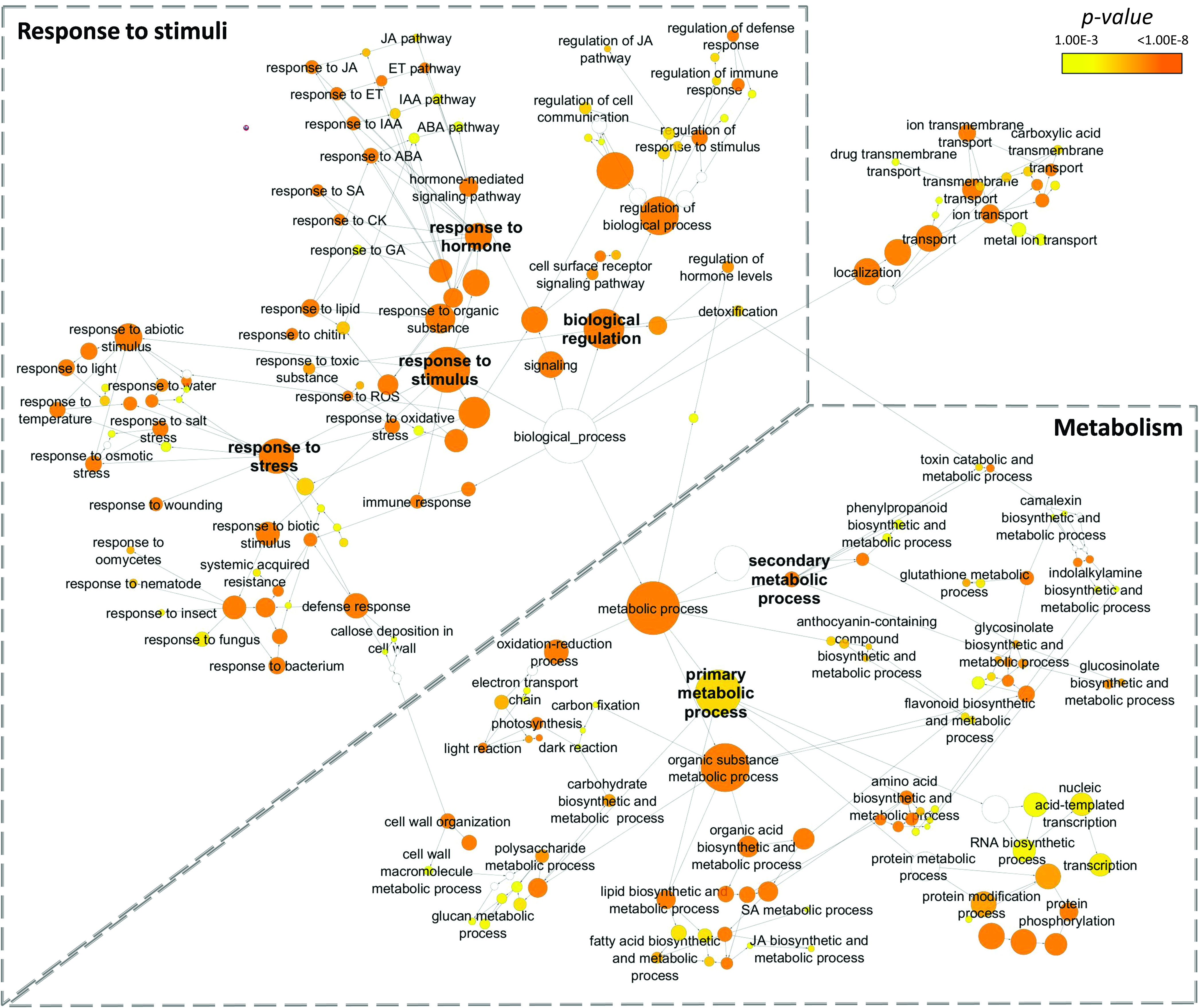

Supplement: FIGURE 2 [file Image_2.JPEG]
