# Supplementary material for: Making a Better Home: Modulation of Plant Defensive Response by Brevipalpus Mites
Source: Front Plant Sci. 2018 Aug 15;9:1147. doi: 10.3389/fpls.2018.01147 (PMC6104575; doi:10.3389/fpls.2018.01147)
Supplement: TABLE 1 [file Table_1.DOCX]

**Supplementary Table 1.** Sequencing and alignment statistics for all *Arabidopsis thaliana* samples. hai: hours after infestation, dai: days after infestation.

| **Samples** | | **Total reads** | | **Mapped reads** | | **Mapped (%)** | | **Uniquely mapped (%)** | **Multimapped (%)** |
| --- | --- | --- | --- | --- | --- | --- | --- | --- | --- |
| 6 hai | Control 1 | | 27341092 | | 25982323 | | 95,0 | 91,8 | 3,2 |
|  | Control 2 | | 41197668 | | 38528164 | | 93,5 | 89,6 | 3,9 |
|  | Control 3 | | 29874712 | | 28303161 | | 94,7 | 91,6 | 3,2 |
|  | Control 4 | | 52661502 | | 49661941 | | 94,3 | 90,9 | 3,4 |
|  | Mites 1 | | 51828306 | | 48872195 | | 94,3 | 91,0 | 3,3 |
|  | Mites 2 | | 53937770 | | 50967558 | | 94,5 | 91,4 | 3,1 |
|  | Mites 3 | | 51890962 | | 49001322 | | 94,4 | 91,3 | 3,2 |
|  | Mites 4 | | 56200284 | | 53208550 | | 94,7 | 91,4 | 3,2 |
| 2 dai | Control 1 | | 26757516 | | 25382928 | | 94,9 | 91,8 | 3,0 |
|  | Control 2 | | 34948300 | | 33097816 | | 94,7 | 91,6 | 3,1 |
|  | Control 3 | | 28828048 | | 27131072 | | 94,1 | 91,1 | 3,0 |
|  | Control 4 | | 43861198 | | 41453036 | | 94,5 | 91,5 | 3,0 |
|  | Mites 1 | | 38755886 | | 36221667 | | 93,5 | 90,7 | 2,8 |
|  | Mites 2 | | 45170082 | | 42526976 | | 94,1 | 91,2 | 2,9 |
|  | Mites 3 | | 50900656 | | 48065714 | | 94,4 | 91,4 | 3,1 |
|  | Mites 4 | | 39623954 | | 36967715 | | 93,3 | 90,4 | 2,9 |
| 6 dai | Control 1 | | 34084010 | | 32319770 | | 94,8 | 91,7 | 3,1 |
|  | Control 2 | | 32375276 | | 30627173 | | 94,6 | 91,8 | 2,8 |
|  | Control 3 | | 31969108 | | 30052446 | | 94,0 | 91,2 | 2,8 |
|  | Control 4 | | 40517334 | | 38180279 | | 94,2 | 91,1 | 3,1 |
|  | Mites 1 | | 41967600 | | 39203750 | | 93,4 | 90,7 | 2,7 |
|  | Mites 2 | | 47551420 | | 44768874 | | 94,1 | 91,4 | 2,8 |
|  | Mites 3 | | 42260866 | | 39386679 | | 93,2 | 90,4 | 2,8 |
|  | Mites 4 | | 50360450 | | 47480937 | | 94,3 | 91,5 | 2,8 |
| Average | | 41.452.666,7 | | 39.058.001,9 | | 94,2 | | 91,2 | 3,1 |
