# Supplementary material for: Making a Better Home: Modulation of Plant Defensive Response by Brevipalpus Mites
Source: Front Plant Sci. 2018 Aug 15;9:1147. doi: 10.3389/fpls.2018.01147 (PMC6104575; doi:10.3389/fpls.2018.01147)
Supplement: TABLE 9 [file Table_9.DOCX]

**Supplementary Table 9**. Selected genes and corresponding primer pairs of *Arabidopsis thaliana* used for gene expression analyses by RT-qPCR.

| Gene | Name | Locus | Sequences (5´-3´) | Reference |
| --- | --- | --- | --- | --- |
| *EXP3* | *Expansin 3* | AT2G37640 | F:AATGCACACGCCACTTTCT  R:ACAAGTTCCCGTACCCACAC | (Verkest et al., 2014) |
| *FBOX* | *F-box family protein* | AT5G15710 | F:GGCTGAGAGGTTCGAGTGTT  R:GGCTGTTGCATGACTGAAGA | (Lilly et al., 2011) |
| *GRX480* | *Thioredoxin superfamily protein* | AT1G28480 | F:GATTGATGAGGAGAGGGAAGATG  R: CTACATAAACCGCCGGTAACT | This work |
| *MYC2* | *Jasmonate insensitive 1* | AT1G32640 | F:AGCAACGTTTACAAGCTTTGATTG  R:TCATACGACGGTTGCCAGAA | (Anderson et al., 2004) |
| *ORA59* | *Octadecanoid-responsive AP2/ERF 59* | AT1G06160 | F:CTTGTTCTCTTTGCTGCTTTCGAC  R:TTGGCTCCTTCAAGGTTAATGCAC | (Czechowski et al., 2004) |
| *PDF1.2* | *Plant defensin 1.2* | AT5G44420 | F:CTTGTTCTCTTTGCTGCTTTCGAC  R:TTGGCTCCTTCAAGGTTAATGCAC | (López et al., 2011) |
| *PR1* | *Pathogenesis-related gene 1* | AT2G14610 | F:GTGCCAAAGTGAGGTGTAACAA  R:CGTGTGTATGCATGATCACATC | (Lindermayr et al., 2010) |
| *PR2* | *Pathogenesis-related gene 2* | [AT3G57260](https://www.arabidopsis.org/servlets/TairObject?id=37029&type=locus) | F:AGCTTCCTTCTTCAACCACACAGC  R:TGGCAAGGTATCGCCTAGCATC | (Lou et al., 2016) |
| *RGL3* | *RGA-like protein 3* | AT5G17490 | F:ATGGATACAGAGTGGAGGAGAACG  R:GATGCAGCGATTAGAGGTTTCG | (Magome et al., 2008) |
| *SAND* | *SAND family protein* | AT2G28390 | F:AACTCTATGCAGCATTTGATCCACT  R:TGATTGCATATCTTTATCGCCATC | (Czechowski et al., 2005) |
| *VSP2* | *Vegetative storage protein 2* | AT5G24770 | F:ACGCAAAATATGGATACGGAACAG  R:CGGTCCCTAACCACAACCAGTA | (Bischoff et al., 2009) |
| *WRKY70* | *WRKY DNA-binding protein 70* | AT3G56400 | F:GGAAGAAGACAATCCTCATCGT  R:CGTTTTCCCATTGACGTAACT | (Von Saint Paul et al., 2011) |
